# Evaluating the accuracy of root transparency and periodontosis age estimation models in a Portuguese population

**DOI:** 10.1007/s12024-025-01000-z

**Published:** 2025-04-07

**Authors:** Viviana Rocha, Flávia Mendes, Maria Lurdes Pereira, Inês Morais Caldas

**Affiliations:** 1 UCIBIO - Research Unit on Applied Molecular Biosciences, Forensic Sciences Research Laboratory, University Institute of Health Sciences (1H-TOXRUN, IUCS-CESPU), Gandra, Portugal; 2 Associate Laboratory I4 hb – Institute for Health and Bioeconomy, , University Institute of Health Sciences – CESPU, 4585 - 116 Gandra, Portugal; 3 Instituto de Ciências Biomédicas Abel Salazar da Universidade Do Porto, Porto, Portugal; 4 Faculdade de Medicina Dentária, Universidade Do Porto, Rua Dr. Manuel Pereira da Silva, 4200 - 393 Porto, Portugal; 5 EPIUnit of Instituto de Saúde Pública da Universidade Do Porto, Porto, Portugal; 6 Laboratory for Integrative and Translational Research in Population Health (ITR) of Instituto de Saúde Pública da Universidade Do Porto, Porto, Portugal

**Keywords:** Forensic science, Dental age estimation, Lamendin method, Fialho method, Prince and Ubelaker method, Tooth wear, Population-specific data

## Abstract

This study aims to evaluate the accuracy of existing dental age estimation models, including the Lamendin, Prince & Ubelaker, Fialho, and modified Fialho methods, within a Portuguese population. Dental techniques, particularly those involving root transparency and periodontosis, are examined due to their relevance in forensic age estimation. A sample of 166 single-rooted teeth from individuals aged 30 to 86 was analyzed. Measurements included root transparency, periodontosis, and tooth and root length. Statistical methods were applied to assess the reliability and accuracy of each model in estimating age, considering the potential influence of environmental and lifestyle factors on dental aging. Root transparency emerged as a robust age indicator, consistently correlating with chronological age. In contrast, periodontosis introduced variability due to external influences, reducing its reliability. The modified Fialho model, which focuses solely on root transparency, showed the highest accuracy, suggesting that eliminating periodontosis from the estimation process may enhance reliability in populations where environmental factors heavily affect dental aging. The findings underscore the importance of population-specific adjustments in dental age estimation models. By refining methods like the modified Fialho model, forensic investigations can achieve more accurate results, particularly in populations where external factors influence periodontosis.

## Introduction

Estimating the age of adults based on dental features is an essential practice in forensic science, particularly in odontology, anthropology, and archeology [1, 2]. Accurately estimating the age at death can provide a necessary understanding of unidentified remains and contribute significantly to individual identification processes and population demographic studies [3].

Estimating chronological age becomes particularly challenging in adults, where skeletal growth has ceased [1]. Unlike juveniles, whose age is often estimated based on developmental stages [2, 4], adults require methodologies focusing on degenerative changes and wear patterns [5–8].

The challenge in narrowing the age range for adults lies in relying on methods primarily based on the degree of bone remodeling or degenerative processes. These processes are highly influenced by biological variability and environmental factors, making precise estimations difficult [9]. Generally, as the age of the individual increases, the estimated age range becomes broader[10]. Consequently, in some cases, only general indications can be provided (e.g., age over 50) using markers such as dental wear [11], maxillary suture obliteration [12, 13], pubic symphyseal surface morphology [7, 14], sternal rib end changes [15, 16], or cranial suture closure [17].

Among the various biological structures used for age estimation, teeth stand out due to their resilience against environmental and taphonomic processes [18], making them one of the most reliable indicators in forensic contexts [19]. Their durability makes them invaluable in highly decomposed or fragmentary remains forensic investigations [18, 19]. Furthermore, dental tissues undergo predictable biological changes with age, including attrition, secondary dentin deposition, periodontal ligament changes, pulp changes, cementum annulations, and root transparency [8, 20–24]. These features form the basis for age estimation methodologies that leverage observable and measurable dental morphology and structure changes. In adult age estimation, several dental methods can be used, some of which can only be applied to the dead, as they require tooth extraction and/or tooth fragmentation. Examples of such methodologies include the analyses of root transparency alone or assessing the periodontal status [25–29], aspartic acid racemization [30], and the assessment of tooth cementum annulations [24].

Non-destructive methods include analyzing occlusal dental wear [31, 32], radiographic visibility of the third molar pulp [21] or periodontal ligament [20], radiographic analyses of secondary dentine deposition [22], and dental color measurement [33].

Still, adult dental age estimation faces several challenges that can affect the accuracy and reliability of the methods [34]. Biological variability is one of those challenges, as the rate of age-related changes in dental tissues varies significantly among individuals due to genetic factors, lifestyle, diet, and environmental influences [35]. This variability complicates the development of universally applicable models. Furthermore, age estimation methods are often developed for specific populations [36–39]. Applying these methods to other populations without validation can lead to significant errors due to differences in dental morphology and wear patterns [40]. Limited accuracy in older adults is another problem since the physiological changes in dental tissues often plateau or display more significant variability as individuals age [41]. This results in broader age ranges and decreased precision in age estimations for older adults. One must also account for the influence of pathological conditions that can alter tooth structure and make it difficult to rely on specific tooth structures used as markers (dental diseases such as caries, periodontitis, restorative treatments, and tooth loss, among others) [41, 42]. Subjectivity in the assessment can also be an issue [42], even with standardized protocols. Other limitations can arise from destructive methods since not all dental techniques are conservative. Histological analysis, for example, provides high accuracy but is only sometimes feasible due to ethical concerns, legal restrictions, or the need to preserve the integrity of remains [43]. Non-destructive methods, such as radiographic analysis, are less invasive but may provide a different level of detail, potentially reducing accuracy, and can also be limited by ethical concerns [44]. Furthermore, these methods often require access to specialized equipment and expertise, which may only sometimes be available.

Moreover, although dental structures resist environmental factors, they are not indifferent. Habits like smoking, chewing tobacco, or abrasive diets can accelerate or alter dental wear [8, 45], introducing further variability into age estimation. In methods like Lamendin's, the periodontal recession is a key variable [41]. However, it is affected by age and external factors, such as oral hygiene practices and systemic health, complicating its use as a reliable indicator.

As with other methodologies, many methods are validated using small or non-representative samples, limiting their generalizability. To enhance reliability, more extensive studies that include diverse populations are needed [2, 3, 46].

Finally, another difficulty is isolating age-related changes. In adults, distinguishing changes solely due to aging from those influenced by external factors or individual variability is inherently challenging.

Among the myriad methods developed for dental age estimation, several have become particularly prominent due to their widespread application and reported accuracy.

**Fig. 1:**
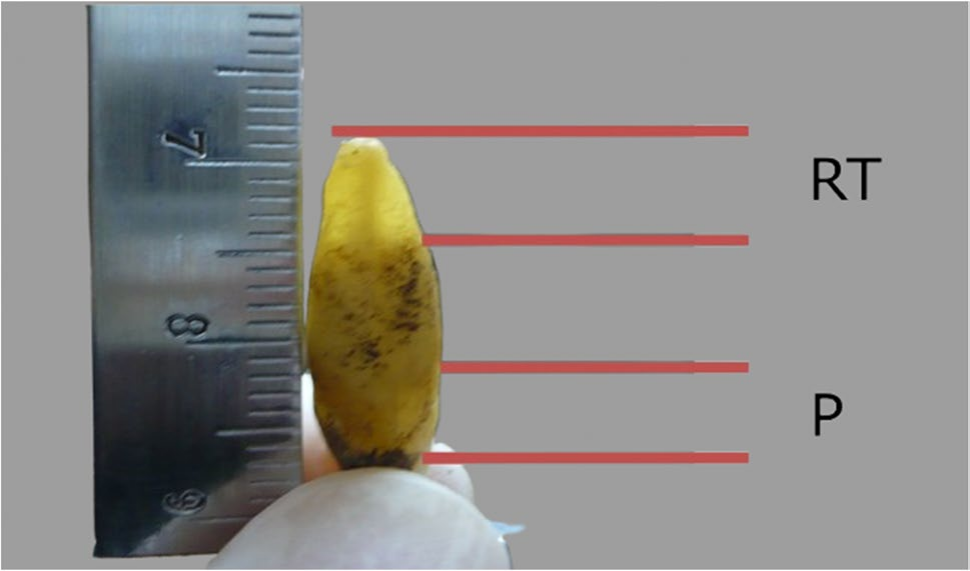
Measurement of periodontal height (P) and translucency height (RT)

For example, the methods developed by Gustafson and Lamendin have been widely used for decades. Gustafson’s method evaluates six dental variables, including attrition, secondary dentin, and cementum apposition, assigning scores to estimate age [47]. Lamendin’s technique simplifies this process by focusing on periodontal recession and root transparency (Fig. 1), offering a rapid and practical alternative [48]. The method provides a formulaic approach, combining these variables with a constant derived from empirical data. It is suitable for both sexes and applies to all types of single-rooted teeth. Although widely used, its accuracy can vary significantly across different populations, indicating a need for localization and adaptation [36].

Prince and Ubelaker [27] applied this technique to the Terry Collection to assess the accuracy of Lamendin's method. The authors obtained suitable results with comparable, sometimes lower mean errors than those of Lamendin and co-workers. They noted that ancestry and especially sex should be considered when estimating age-at-death using root translucency and periodontosis. Other studies have supported this proposal [36, 49].

Fialho's method [50], introduced in 2016, addresses some of these concerns by adapting the original Lamendin technique to a Portuguese population, suggesting a more localized approach to age estimation. Furthermore, the modified Fialho method simplifies the estimation process by focusing solely on root transparency, aiming to streamline the procedure but potentially at the cost of reducing multi-factorial accuracy.

While the robustness and adaptability of Lamendin's technique have been demonstrated in multiple populations, these methods are not without limitations. The potential influence of population-specific factors, such as differences in diet, lifestyle, and genetics, requires careful consideration when applying the technique across diverse demographic groups [3, 37, 38, 40, 51]. Several studies emphasize the need for validation and, when necessary, the development of population-specific formulae to ensure accuracy. Thus, although Lamendin’s method is broadly applicable, ongoing research continues to refine its use and address the subtle demographic variables that may affect precision.

Recent technological advancements have further refined dental age estimation methods. Digital imaging, machine learning, and high-resolution micro-computed tomography have enhanced the accuracy and objectivity of analyses. These approaches allow for the quantification of dental characteristics with unprecedented precision, facilitating the development of population-specific models and reducing observer bias.

Despite these advancements, challenges remain. Disease, dental restoration, and individual variability can obscure age-related changes, complicating the estimation process. Furthermore, while radiographic and histological methods are highly accurate, they often require specialized equipment and expertise, limiting their accessibility in resource-constrained settings.

This study focuses on refining adult dental age estimation techniques by analyzing key dental features. By examining the applicability of some existing methods, this research aims to develop more reliable, reproducible, and population-specific models. Ultimately, these efforts enhance the precision of forensic age estimation and broaden its utility in diverse investigative contexts.

With this work, we’ve aimed to test the age estimation formulae proposed by Lamendin et al. (1992), Prince and Ubelaker (2002), Fialho (2016), and modified Fialho (2016) in a modern Portuguese sample. Moreover, a new model for age estimation in adults was proposed.

## Materials and methods

### Study design and sample selection

This study analyzed 166 single-rooted teeth from individuals aged between 30 and 86 years (mean age: 59.64 years, median: 60.00 years, standard deviation: 13.62). Of the participants, 112 (67.5%) were male (mean age: 56.73 years, median: 58.00, standard deviation: 14.61), and 54 (32.5%) were female (mean age: 65.67 years, median: 67.00, standard deviation: 8.69). The ages ranged from 30 to 80 years for males and 47 to 86 years for females.

The individuals were all Portuguese nationals drawn from the Identified Skeletons Collection of the University Institute of Health Sciences (IUCS) [52]. Portugal is considered one of the most ethnically homogenous countries, with approximately 95% of the population being ethnic Portuguese.

### Inclusion and exclusion criteria

Inclusion Criteria: 1) Portuguese nationality; 2) Age at death of 30 years or older; 3) Presence of at least one intact single-rooted tooth.

Exclusion Criteria: 1) Teeth with fractures, decay, fillings, or other treatments.

### Methodological calibration

Before data collection, a calibration session was conducted to ensure consistency in measurement techniques. Observers practiced measurements on a subset of teeth to standardize procedures and minimize interobserver and intraobserver variability.

### Measurements and data collection

The following measurements were obtained: 1)Root Transparency (RT): Assessed under a light source; 2) Periodontosis (P): Evaluated visually; 3)Root Length (RL): Measured with a digital caliper accurate to 0.01 mm; 4) Tooth Length (TL): Measured with the same precision as RL.

Root transparency and periodontosis are depicted in Fig. 1.

All measurements were recorded in a Microsoft Excel spreadsheet for analysis.

### Age estimation models

The collected data were tested against the following models:

1. **Lamendin (1992)**: Age = 0.18 × P + 0.42 × T + 25.53 Age = 0.18 × P + 0.42 × T + 25.53
  Where P = Periodontosis × 100Root LengthP = Root LengthPeriodontosis × 100, and T = Root Transparency × 100Root LengthT = Root LengthRoot Transparency × 100​.
2. **Prince and Ubelaker (2002)**: Separate formulas were applied for males and females:
  Males: Age = 0.15 × RH + 0.29 × P + 0.39 × T + 23.17 Age = 0.15 × RH + 0.29 × P + 0.39 × T + 23.17.
  Females: Age = 1.10 × RH + 0.31 × P + 0.39 × T + 11.82 Age = 1.10 × RH + 0.31 × P + 0.39 × T + 11.

RHRH is root length, with PP and TT calculated as per Lamendin's method.

3. **Fialho (2016):** Adapted for Portuguese populations:
  Age= 2.518+PRL× 8.308+TRL× 85.878 Age= 2.518+RLP× 8.308+RLT× 85.878
4. **Modified Fialho (2016)**: Solely for root transparency:
  Age= 32.645+TRL× 75.558 Age= 32.645+RLT× 75.558

### Statistical analysis

Data analysis was conducted using SPSS Version 29.0. The following statistical approaches were employed:

Reliability Testing: A random subset of 20 teeth was measured twice by the same observer (one week apart) and by a second observer. Intraclass and interclass correlation coefficients (ICCs) were calculated to assess repeatability and reproducibility. According to Fleiss [53], ICC values were interpreted as follows: below 0.4 = poor reliability; 0.4–0.75 = moderate to good reliability; above 0.75 = excellent reliability. The coefficients for each feature were:

- Tooth Length: Displays the highest reliability, with an intraclass ICC of 0.988 and an interclass ICC of 0.980, indicating nearly perfect agreement.
- Root Length: Also demonstrates strong reliability, with an intraclass ICC of 0.912 and an interclass ICC of 0.901.
- Root Transparency: Shows slightly lower but still high reliability, with an intraclass ICC of 0.901 and an interclass ICC of 0.888.
- Periodontosis: Has the lowest reliability among the four parameters, with an intraclass ICC of 0.853 and an interclass ICC of 0.853, though it remains within acceptable levels of agreement.

Overall, the data indicate excellent reliability for tooth length, root length, and root transparency, while periodontosis measurements show slightly reduced but acceptable consistency. These ICC values suggest that the methods for measuring these parameters are reliable for intra- and inter-observer agreement.

**Descriptive Statistics**: Categorical variables were expressed as absolute frequencies and percentages, while continuous variables were summarized as minimum, maximum, mean, and standard deviation.

**Inferential Statistics**: The choice of parametric tests was based on the assumption of normality supported by the Central Limit Theorem, given the sample size. Although minor deviations from normality may exist, the sample size was considered sufficient to approximate a normal distribution, allowing the use of parametric statistical methods. The steps taken were the following:

- Paired t-tests assessed differences between chronological and estimated ages.
- Pearson test analyzed correlations between variables.
- A linear regression model was developed for age estimation.

The significance level was set at 5%.

## Results

In the initial phase, given that Price and Ubelaker's models identify sex as a variable of interest, the application of various models was studied from this perspective.

Table 1 shows males'mean chronological and estimated age differences (calculated using different methods). Regardless of the method used, there were consistently statistically significant differences between the chronological and estimated ages. In males, the Price and Ubelaker method showed the highest mean difference between the EA and CA (26.65 years), followed by Lamendin (17.31 years), Fialho (6.14 years), and Modified Fialho (4.02 years). All methods demonstrate statistically significant differences between EA and CA with *p*-values < 0.05.

**Table 1:** Difference between mean chronological age and estimated age, for men, in years (Statistically significant differences in bold)

Method	Mean difference	SD	Standard error of mean	95% CI		t	df	*p*
				Upper	Lower			
Lamendin	17.31	13.54	1.28	14.77	19.84	13.53	111	** < 0.001**
Fialho	6.13	15.14	1.43	3.30	8.97	4.29	111	** < 0.001**
Modified Fialho	4.02	15.58	1.38	1.29	6.74	2.92	111	**0.004**
Prince & Ubelaker	26.65	28.17	2.66	21.40	31.93	10.02	111	** < 0.001**

*SD* Standard deviation; *CI* Confidence interval; *T* Test t; *DF* Degrees of freedom

Table 2 shows females'mean chronological and estimated age differences (calculated using different methods). Regardless of the method used, there were consistently statistically significant differences between the chronological and estimated ages.

**Table 2:** Difference between mean chronological age and estimated age, for females, in years (Statistically significant differences in bold)

Method	Mean difference	SD	Standard error of mean	95% CI		t	df	*p*
				Lower	Upper			
Lamendin	25.08	10.00	1.36	22.35	27.81	18.43	53	** < 0.001**
Fialho	15.20	12.81	1.74	11.71	18.70	8.72	53	** < 0.001**
Modified Fialho	13.71	11.73	1.59	10.51	16.91	8.59	53	** < 0.001**
Prince & Ubelaker	34.55	8.81	1.98	32.15	36.96	28.84	53	** < 0.001**

*SD* Standard deviation; *CI* Confidence interval; *T* Test t; *DF* Degrees of freedom

Results were similar to those obtained in males, with the Price and Ubelaker method showing the highest mean difference between the EA and CA (34.55 years), followed by Lamendin (25.08 years), Fialho (15.20 years), and Modified Fialho (13.71 years). Once again, all methods demonstrate statistically significant differences between EA and CA with *p*-values < 0.05.

As for the differences between estimated age and chronological age in the different age groups, in men, in the age group 30–39 years, estimated age by the Lamendin method was the only one not displaying statistically significant differences. In the remaining groups, Lamendin and Prince & Ubelaker’s methods consistently underestimated age across all age groups. The Fialho method overestimated age in younger groups (ages 30–39 and 40–49) and underestimated age in the remaining groups, displaying no differences between estimated and chronological age for groups 40–49 (*p* = 0.369 and *p* = 0.056, respectively) and 50–59 (*p* = 0.813 and *p* = 0.482, respectively). Lastly, the Modified Fialho method also overestimated age in the younger groups (ages 30–39; 40–49; and 50–59) and underestimated age in the remaining groups, displaying no differences between estimated and chronological age for groups 40–49 (*p* = 0.056) 50–59 (*p* = 0.482), and 70–79 (9 = 0.06) (Table 3).

**Table 3:** Mean estimated age (MEA) by group, in years (y), in males, using Lamendin, Prince & Uberlaker (P&U), Filho and Modified Fialho (p value refers to the difference between mean chronological and mean estimated age)

Methods	Age 30–39 (mean 34.83y)		Age 40–49 (mean 46.73y)		Age 50–59 (mean 53.88y)		Age 60–69 (mean 62.52y)		Age 70–79 (mean 73.97y)		Age 80–89 (mean 80.00y)	
	MEA	*p*	MEA	*p*	MEA	p	MEA	*p*	MEA	*p*	MEA	*p*
Lamendin	34.44	0.646	38.45	< 0.001	40.31	< 0.001	39.95	< 0.001	42.61	< 0.001	37.73	< 0.001
P&U	26.92	0.018	27.33	< 0.001	38.24	0.139	27.69	< 0.001	28.60	< 0.001	26.78	0.004
Fialho	41.31	< 0.001	49.18	0.369	53.33	0.813	52.06	< 0.001	55.17	< 0.001	46.97	0.061
Modified Fialho	44.78	0.002	51.62	0.056	55.35	0.482	54.10	< 0.001	56.25	< 0.001	49.48	0.06

As for females, both Lamendin and Prince & Ubelaker’s methods underestimated age across all age groups, with significant differences in all age groups, except for the older group (age > 80 years), where no statistically significant differences were found between Lamendin’s estimated and chronological age. The Fialho and modified Fialho method underestimated age in all groups, with statistically significant differences (*p* < 0.001) for all groups except 80–89 (*p* = 0.373 and *p* = 0.283, respectively) (Table 4).

**Table 4:** Mean estimated age (MEA) by group, in years (y), in females, using Lamendin, Prince & Uberlaker (P&U), Filho and Modified Fialho (*p* value refers to the difference between mean chronological and mean estimated age)

	Age 50–59 (mean 54.60y)		Age 60–69 (mean 62.45y)		Age 70–79 (mean 72.95y)		Age 80–89 (mean 86.00y)	
	MEA	*p*	MEA	*p*	MEA	*p*	MEA	*p*
Lamendin	38.62	< 0.001	43.24	< 0.001	47.32	< 0.001	53.80	0.075
P&U	30.91	< 0.001	30.59	< 0.001	31.92	< 0.001	30.89	0.016
Fialho	47.42	< 0.001	54.32	< 0.001	45.31	< 0.001	73.35	0.373
Modified Fialho	49.54	0.003	54.95	< 0.001	47.71	< 0.001	73.31	0.283

Analyzing the correlation between the various variables under study and age, it was found that the maximum tooth length showed a moderate positive correlation with age (r = 0.539), indicating that as age increases, this variable tends to increase correspondingly. The correlation is statistically significant (*p* < 0.001). As for the periodontosis coefficient, a positive correlation with age (r = 0.418) is displayed, suggesting a moderate relationship. This result is also statistically significant (*p* < 0.001). Root translucency exhibited a weaker positive correlation with age (r = 0.300) but remains statistically significant (*p* < 0.001). Root length demonstrated an almost perfect positive correlation with age (r = 0.992), showing that root length is a highly reliable predictor of age. This relationship is significant with a slightly different threshold (*p* = 0.001). Finally, the variable sex had a weak positive correlation with age (r = 0.308), suggesting a slight association, and this correlation is statistically significant (*p* < 0.001).

The maximum tooth length and root length were, unsurprisingly, strongly correlated (0.817, *p* < 0.001), and only the maximum tooth length had a significant correlation with age; therefore, only the maximum tooth length was retained for constructing a predictive model. Similarly, the correlation of the periodontosis coefficient with root translucency (0.190, *p* = 0.014) was also observed, leading to the exclusion of this variable from the model.

Thus, tooth length, root translucency, and sex were used. The necessary assumptions for constructing a linear regression model were then verified: linearity in the relationship between variables, independence and homoscedasticity of errors, normality of errors, absence of multicollinearity among independent variables, and relevance of predictors. An R^2^ of 0.529 was obtained, which, in other words, explains 52.3% of the observed variance. The equation follows Age = 61.45 + 0.965 TL—1.472 TR—7.276S, With TL representing the maximum tooth length, TR the root translucency, and S, the sex (1 = male; 2 = female).

## Discussion

Age estimation in adults using dental techniques is a complex subject. Only a few papers on this subject exist in the Portuguese population, and most address the pulp/tooth ratio in the lower second premolar [37], the upper and lower canines [38], and the central incisors [22]. The dental pulp's and periodontal ligaments'third molar radiographic visibility were also assessed [20, 21]. As for studies regarding root translucency and periodontosis, we could only find one from Fialho [50], making the need for more investigation in this field evident.

Our research highlights significant discrepancies between forensic models’ chronological and estimated ages, underscoring the need for methodological refinement and population-specific adaptation.

The Lamendin method, which combines root translucency, periodontosis, and root length for age estimation, demonstrated limitations, especially in male subjects in older subjects, where the mean underestimation of age exceeded 50 years (MEA = 37.73 vs. mean chronological age = 80.00; *p* < 0.001), and in females, in younger groups (50–59 years: MEA = 38.62; *p* < 0.001). Moreover, regardless of sex, this method consistently underestimated the MEA in all age groups, except for males, in the age group 30–39 years (*p* = 0.646), suggesting that the Lamendin method may not be reliable for older individuals.

This outcome aligns with the findings of other researchers, such as Foti et al. [42], who noted that population-specific variables, like genetic and environmental factors, may limit the Lamendin method’s accuracy.

However, our results don’t agree with recent studies that have refined Lamendin's method [54, 55]. In these studies, the authors use the same variables proposed by Lamendin et al., i.e., the maximum root length, the periodontal recession, and the root dentine translucency, and incorporate Bayesian algorithms and additional statistical techniques. This enhances the method's applicability across diverse populations while maintaining accuracy within 10 years, especially for individuals aged 30–60. The differences between these methodologies may explain the differences.

Moreover, the method's accuracy was corroborated in South African populations, where biological variations further contributed to its reduced effectiveness [36]. Multiple studies have consistently supported the centrality of root translucency as a robust indicator of age. Lamendin et al. [48] initially established root translucency as a reliable dental trait for age estimation, with the translucency increasing with age due to the deposition of hydroxyapatite crystals within the dentin tubules [26]. This natural process progresses from the root apex towards the cervix, and its correlation with age has been confirmed across various populations [56]. However, as pointed out by Gibelli et al. [19], environmental exposure, particularly to high temperatures or specific soil compositions, can alter root translucency, complicating its application in archaeological contexts. Despite these environmental factors, root translucency remains unaffected mainly by individual lifestyle choices like diet and oral hygiene [5], making it a reliable marker in controlled forensic contexts.

On the other hand, periodontosis presents more variability, as it is influenced by extrinsic factors such as diet, dental hygiene, and physical or mechanical irritation [27, 42]. Prince and Konigsberg [56] further support this, emphasizing that individuals'lifestyle and health status can lead to inconsistencies when using periodontosis as an age estimator. This variability was reflected in our study, where periodontosis coefficients showed a lower correlation with age than root translucency, suggesting that periodontosis should be applied cautiously, especially in heterogeneous populations.​

The modified Fialho method focuses solely on root translucency. Roberts et al. [11] referred to the Bang and Ramm method [29] as the gold standard in forensic odontology for age estimation, utilizing root translucency as an age proxy. This method is based on the premise that the dentinal tubule lumens gradually become occluded with mineral deposits over time, increasing light scatter within the root [23]. This process starts at the root apex and progresses coronally as the individual ages [7]. Several studies have presented results, with Tang's et al. research [25] reporting an average absolute difference between age and estimated age of 10.7 years and 8.4 years using the Bang and Ramm method. These authors also noted that age was overestimated in younger and underestimated in older individuals.

This method demonstrated higher accuracy in our sample, yet the limitations of single-factor models remain evident. The exclusion of multi-factorial variables, while simplifying the process, potentially reduces the precision of age estimation. This reflects broader critiques in forensic anthropology, where demographic and lifestyle variables can introduce considerable error if not adequately controlled [49]. The high correlation between tooth wear and age supports the inclusion of tooth length as a dynamic indicator of aging, complementing static measurements like root translucency and root length [11]​. The findings from our study further underscore the need for continuous calibration of age estimation models. As proposed by Lamendin et al. [48], incorporating multiple dental traits improves the reliability of age estimates, but the influence of population-specific factors remains a significant challenge. For future studies, adjustments to the Lamendin formula could involve greater localization, as indicated by Prince and Ubelaker [27], who successfully adapted the method for diverse skeletal samples, reducing the mean error when accounting for ancestry and sex​.

We divided our sample using a 10-year interval gap because it represents a balance between precision and inclusiveness in age estimation. A range of 10 years is generally reliable for forensic purposes, and very narrow ranges may exclude potential candidates from missing persons lists, reducing the chances of successful identification. Excessively wide ranges may include unrelated individuals, complicating and delaying the identification process [54]. Using a 10-year interval, we aim to provide an age range that is practical and effective for narrowing down potential matches without compromising accuracy.

Current literature [36, 54, 55] suggests a trend toward underestimation in the older age group, indicating that root translucency would cease to be helpful as a good estimator for individuals older than 60. In our investigation, in males, the Lamendin method performed exceptionally well in the 30–39 age group, estimating a mean age (MEA) of 34.44 years, which is remarkably close to the chronological mean age of 34.89 years. This demonstrates the method's strong reliability and accuracy for this age group. A similar level of performance was observed only in the 50–59 age group for males, but with the Fialho and Modified Fialho methods, which estimated MEAs of 53.33 years and 55.35 years, respectively. These estimations closely align with this group's chronological mean age of 53.88 years. Such accurate predictions highlight the potential of these methods when applied to specific age groups, although their performance is inconsistent across all groups. This reinforces the importance of tailoring the selection of methods to the demographic characteristics of the individuals being evaluated.

The absence of statistically significant differences in the 80–89 age group for the Fialho and Modified Fialho methods should be interpreted cautiously. This group likely had a small sample size, which may introduce bias or reduce the statistical power to detect significant differences. Consequently, these results may not fully represent the performance of these methods in estimating age for this group.

Generally, all methodologies presented fewer discrepancies between EA and CA in males, possibly due to the difference in sample size. The literature shows diverging results as to the technique's correlation with sex; some authors found better results for males [42, 57], others observed better estimates for females [58], while other studies reported no difference [27].

Our model relies on root translucency, a variable that, as explained, presents some advantages over periodontosis. We’ve also incorporated tooth length over root length. In forensic age estimation, tooth length and root length have been explored as key variables, each offering unique advantages and disadvantages. In our study, tooth length emerged as a more reliable indicator of age than root length, mainly when used with root translucency.

There are several advantages of using tooth length. Tooth length is dynamic and changes over time due to wear, making it a progressively responsive variable in age estimation. As individuals age, natural wear and tear on the teeth can be consistently measured [8, 31, 32, 59, 60]. This predictable wear makes tooth length a practical and reliable indicator of age progression in forensic contexts. Berbesque et al. [6] noted that dietary habits significantly influence tooth wear, which correlates predictably with age, providing a consistent basis for estimating age​.

Additionally, tooth length measurement is relatively straightforward and less susceptible to preservation issues than root length, especially in archaeological or forensic cases involving older remains [61]. As teeth are exposed and more straightforward to measure without significant destruction, this allows for more accurate assessments even when skeletal remains are damaged.

However, tooth length also has some limitations. It is highly influenced by external factors such as dietary habits, dental hygiene practices, and individual or population-specific mastication patterns [62]. This variability can introduce errors when applying general age estimation models across different populations. For instance, populations with diets that lead to accelerated tooth wear may have shorter tooth lengths at younger ages, complicating age estimations if these variables are not adequately accounted for. As noted by Foti et al., this variability requires adjustments or the development of population-specific models [42].

Moreover, tooth length may be significantly reduced in individuals with severe dental attrition or those who have undergone dental treatments, such as restorations or crowns. These interventions can artificially affect tooth length, reducing its effectiveness as an age estimator unless such factors are accounted for during the evaluation.

Root length, by contrast, tends to remain more stable after the tooth fully erupts and develops, making it less susceptible to external environmental or lifestyle factors than tooth length. This stability can offer a more consistent marker for age estimation, especially when dental wear is excessive or uneven. Root length is less affected by individual or population-specific factors, making it a potentially more neutral trait in forensic assessments [63]. Despite these advantages, root length has its limitations. Once fully developed, root length remains relatively static, which can diminish its utility in forensic cases where more dynamic indicators of aging are needed. Root translucency and other root characteristics correlate with age, and due to the static nature of root length post-eruption, they are less reliable as a standalone age predictor [64]​.

It is essential to address the composition differences in our sample, particularly regarding sex distribution and age structure. As noted, the female subsample was considerably older than the male subsample, with differences of 11 years in median age and 8.94 years in mean age. This age disparity likely contributed to the higher mean estimation errors observed in females across all methods. This outcome is consistent with the well-documented tendency for forensic age estimation methods — including Lamendin’s and its derivatives — to show increased error margins in older age cohorts [22, 41, 65]. Table 3 clearly illustrates that from age 60 onwards, all methods systematically underestimate chronological age. Given that half of the female sample is over 61 years old, it is reasonable to attribute part of the higher error observed in female individuals to the age composition of this subsample. Therefore, the observed discrepancy does not necessarily reflect sex-related methodological bias but rather highlights the impact of age distribution in the sample. This bias is an inherent limitation of working with heterogeneous and unbalanced samples, which we acknowledge and discuss. Future studies should aim for more balanced sample structures and may benefit from age-stratified formula adjustments to enhance accuracy in older age groups. Nevertheless, these findings reinforce the need for caution when applying standard models to older individuals and underline the importance of considering both sex and age distribution when interpreting estimation errors.

## Conclusion

In conclusion, the discrepancies observed across age estimation methods highlight the need for population-specific calibrations. The root translucency is a reliable age indicator, especially in modern forensic contexts where environmental and lifestyle factors can be controlled. However, including other dynamic traits like tooth length can improve accuracy, providing a more holistic approach to dental age estimation in forensic investigations.

## Key points

1. Root transparency consistently emerged as the most reliable dental feature for age estimation, strongly correlating with chronological age across various models.
2. Periodontosis was found to be less reliable due to its susceptibility to environmental and lifestyle factors, introducing significant variability in age estimations, especially in heterogeneous populations.
3. Compared to other models, the modified Fialho method, which focuses solely on root transparency, provided the highest accuracy for age estimation within the Portuguese population.
4. Traditional models like Lamendin and Prince & Ubelaker demonstrated significant differences between estimated and chronological ages, particularly for female subjects, where underestimation was substantial.
5. Tooth length, when combined with root transparency, improved the accuracy of age estimations, indicating that dynamic traits like tooth wear contribute to more precise forensic age assessments.

## Data Availability

Data are available upon reasonable request to the corresponding author.
